# Gas exchange and chlorophyll fluorescence responses of *Camellia sinensis* grown under various cultivations in different seasons

**DOI:** 10.1186/s40529-024-00416-0

**Published:** 2024-03-22

**Authors:** Chung-I Chen, Kuan-Hung Lin, Meng-Yuan Huang, Chih-Kai Yang, Yu-Hsiu Lin, Mei-Li Hsueh, Li-Hua Lee, Shiou-Ruei Lin, Ching-Wen Wang

**Affiliations:** 1 Department of Forestry, National Pingtung University of Science and Technology, Pingtung County, 91201 Taiwan; 2 Department of Horticulture and Biotechnology, Chinese Culture University, Taipei City, 11114 Taiwan; 3 Department of Life Sciences and Innovation and Development Center of Sustainable Agriculture, National Chung Hsing University, Taichung City, 40227 Taiwan; 4 Taiwan Biodiversity Research Institute, Nantou County, 552 Taiwan; 5 Tea and Beverage Research Station, Taoyuan City, 326 Taiwan

**Keywords:** Chlorophyll fluorescence, Photosynthetic capacity, Tea plant, Sod culture

## Abstract

Sod culture (SC) and conventional agriculture (CA) represent two distinct field management approaches utilized in the cultivation of tea plants in Taiwan. In this study, we employed gas exchange and chlorophyll fluorescence techniques to assess the impact of SC and CA methods on the photosynthetic machinery of *Camellia sinensi*s cv. TTES No.12 (Jhinhsuan) in response to variable light intensities across different seasons. In spring, at photosynthetic photon flux densities (PPFD) ranging from 800 to 2,000 μmol photon m^-2^ s^-1^, the net photosynthesis rate (Pn, 10.43 μmol CO_2_ m^-2^ s^-1^), stomatal conductance (Gs, 126.11 mmol H_2_O m^-2^ s^-1^), electron transport rate (ETR, 137.94), and ΔF/Fm’ and Fv/Fm (50.37) values for plants grown using SC were comparatively higher than those cultivated under CA. Conversely, the non-photochemical quenching (NPQ) values for SC-grown plants were relatively lower (3.11) compared to those grown under CA at 800 to 2,000 PPFD in spring. Additionally, when tea plants were exposed to PPFD levels below 1,500 μmol photon m^− 2^ s^− 1^, there was a concurrent increase in Pn, Gs, ETR, and NPQ. These photosynthetic parameters are crucial for devising models that optimize cultivation practices across varying seasons and specific tillage requirements, and for predicting photosynthetic and respiratory responses of tea plants to seasonally or artificially altered light irradiances. The observed positive impacts of SC on maximum photosynthetic rate (Amax), Fv/Fm, Gs, water-use efficiency (WUE), and ETR suggest that SC is advantageous for enhancing the productivity of tea plants, thereby offering a more adaptable management model for tea gardens.

**Supplementary Information:**

The online version contains supplementary material available at 10.1186/s40529-024-00416-0.

## Introduction

Tea (*Camellia sinensis* L.) stands as the most widely consumed natural non-alcoholic beverage worldwide, renowned for its rich content of polyphenolic compounds and specialized metabolites (Zeng et al. 2019; Zhang et al. 2020), which are deemed beneficial for human health (Hayat et al. 2015). Representing a lucrative cash crop, the global tea harvest amounted to 5.81 million tons, valued at approximately US $50 billion in 2018 (International Tea Committee, ITC 2021). Specifically, in Taiwan, the tea yield reached approximately 14,341 kg, corresponding to a value of about US $0.4 billion in 2022 (Agriculture and Food Agency, COA, 2023). One particular cultivar, *Camellia sinensis* cv. TTES No.12 (Jhinhsuan), stands out as a globally acclaimed Oolong tea variety.

Conventional agriculture (CA) involves the use of various pesticides, including herbicides, fungicides, and insecticides, in farming operations. This practice often results in pesticide residues in the soil, pollution of natural resources, a reduction in biodiversity, and adverse impacts on food security (Ferdous et al., 2021; Riedo et al., 2021). On the other hand, sod culture (SC) is a form of conservation agriculture that promotes sustainable development by enhancing soil organic matter content, sequestering organic carbon, improving soil physicochemical properties, increasing soil water-holding capacity, advancing microorganism growth and biodiversity, increasing crop quality, reducing disease and pest damage, and improving the garden microclimate (Wang et al. 2016; Zou et al. 2016; Bai et al. 2017; Lin et al., 2019). However, there is a lack of information on the impact of CA and SC on the photosynthetic physiology of tea fields across different seasons and under varying light intensities. Tea plants are extensively cultivated in many countries, but they frequently encounter challenges related to both high and low irradiance throughout their life cycle and growth process. This variability in light intensity affects the geographical distribution of tea plants and significantly limits tea yield and quality (Liu et al. 2017; Dai et al. 2015; Kfoury et al. 2018; Wen et al. 2020; Zhang et al. 2014, 2018, 2020; Xiang et al. 2021). Therefore, understanding the photosynthetic characteristics of tea plants and identifying effective techniques to improve their tolerance to high and low light intensities are crucial for optimizing field cultivation and management practices.

Light is a key environmental signal that triggers chlorophyll (Chl) biosynthesis and induces variable photosynthetic responses based on changes in irradiance (Wang et al. 2021). Chlorophyll fluorescence (ChlF) is a noninvasive technique that accurately measures the functioning of photosynthetic apparati in plants and is frequently used to investigate plant responses to various environmental stresses, both in controlled environments and in the field (Kałuzewicz et al. 2018). ChlF values can provide insights into several aspects of photosynthesis, including the potential for photosynthesis, photochemical dissipation, the percentage of photosystem II (PSII) that is open, the effectiveness of PSII in capturing photo energy from light-harvesting complexes, and the subsequent transfer of quanta (Moya et al. 2019). Variations in light intensity directly impact ChlF values in tea plants, enabling the assessment of the status of their photosynthetic apparatus and photoreceptors. This internal physiological response is recorded as a photosynthetic index, which includes variables such as stomatal conductance (Gs), net photosynthetic rate (Pn), dark respiration rate of CO_2_ (Rd), light quantum yield of CO_2_ (Qy), light compensation point (LCP), and maximum net assimilation of CO_2_ (Amax). These variables are crucial indicators of plant growth and metabolism influenced by climatic factors, such as light intensity (Chen et al. 2021).

In habitats with full sun exposure, leaves often absorb more photons than they can utilize, leading to a reduction in the photochemical efficiency of PSII in plants due to the excess absorbed energy. High irradiance may cause photoinhibition, characterized by a loss of PSII activity and a light-dependent reduction in the fundamental quantum yield of photosynthesis. This requires the dissipation of excess excitation energy (Portela et al. 2019). Non-photochemical quenching (NPQ) is crucial for photoprotection as it quenches excess energy and safely dissipates it as heat (Murchie and Niyogi 2010). A higher NPQ serves as a protective mechanism against photoinhibition and photo-oxidation damage (Feng et al. 2002). Additionally, plants exposed to strong light often exhibit decreases or adjustments in their leaf photosynthetic pigment contents, which is an important photo-protective mechanism (Souza et al. 2017).

In a previous study, we found that an increase in Pn enhanced the positive effects of Amax, maximum quantum efficiency of PSII photochemistry (Fv/Fm), Gs, water use efficiency (WUE), electron transport rate (ETR), and non-photochemical quenching (NPQ) in SC citrus during spring (Chen et al. 2021). This indicates that elevated Pn can benefit citrus production and increase resilience to stress under SC in the central region of Taiwan, considering future climate scenarios. It is essential to quantify adjustments in Pn, Amax, Fv/Fm, Gs, WUE, ETR, and NPQ in response to seasonal variations in solar illumination and tillage management, as citrus leaves release significant amounts of CO_2_ and water. ETR is a rapid method to assess the photosynthetic capacity of citrus under light intensity stress (Chen et al. 2021). It allows for simple evaluations of photosynthesis and estimations of the relationships between heat quenching and photosynthetic efficiency. Variable photosynthesis parameters are sensitive indicators of the physiological status of tested plants and provide a quick means to identify their physiological condition (Wang et al. 2021).

Therefore, we hypothesized that the photosynthetic characteristics would exhibit significant differences between CA and SC over varied seasons and photosynthetic photon flux densities (PPFD) in tea fields. The objectives of this study were to clarify the effects of tillage and seasonal dynamics on the photosynthetic apparatus of tea plants by measuring their photosynthetic capacity and ChlF parameters in response to varying light intensities. Understanding these effects will allow us to evaluate seasonal physiological changes in tea plants and provide tools for improving photosynthetic productivity and planting management patterns of tea plants.

## Materials and methods

### Site description, experimental field management, and experimental design

Tea plants [*Camellia sinensis* L. O. Kuntze cv. TTES No.12 (Jhinhsuan)] 45–55 cm tall and more than 20 years old were grown in two experimental fields, one under CA (120°39’12.4”E, 23°38’36.7”N, Figure S1A) and the other under SC (120°39’06.2"E, 23°38’26.3"N, Figure S1B) at certified organic plantations grassland covers more than 80% of the area (Liu et al. 2021). CA and SC practices for controlling soil and water losses were similar from June, 2019, to May, 2020. Both plantations are located in a low-elevation (ca. 400 m) mountainous area of central Taiwan where the climate is humid subtropical. Mean annual rainfall of 2,500 mm and mean annual air temperature of 24.5 °C were recorded from June 2019 to May 2020 (Figure S2). The world reference base for soil resources classifies its soil as a typical andosol in which the texture of the upper surface is sandy loam.

The experiment took place in the two agricultural systems over four seasons (spring was from March to May, summer was from June to August, fall was from September to November, and winter was from December to February), with each cover crop treatment having four replications. The cover crops were *Paspalum conjugatum* Berg., *Cynodondactylon* (L.) Pers., *Wedelia chinensis* Merr., and *Bidens pilosa* var. *pilosa*. CA fields were cropped with a flail mower in the first week of each season, with residues being left on the soil surface for nutrient supply. Organic cultivation methods were performed, including no chemical fertilizers or herbicides being used during the experiment. No severe pests or diseases were encountered during the experiments. A completely randomized design employing the two no-tillage methods and four seasonal treatments was used, and there were five replicates per treatment. For each treatment, five intact, fully expanded leaves (the second to third mature leaves) and leaf samples were randomly sampled from robust and healthy plants at the end of each season.

### Determination of photosynthetic capacityand ChlF parameters with a fixed light source

In all seasons, PPFD was adjusted to 0, 5, 10, 15, 25, 50, 75, 100, 200, 400, 800, 1200, 1500, 1800, and 2000 μmol photon m^− 2^ s^− 1^ in a leaf chamber for 75 min to understand how radiant energy was used by the tested plants under different illumination intensities. Plants were measured with a gas-exchange and fluorescence photosynthesis analyzer (GFS-3000FL, Walz, Effeltrich, Germany) from June 2019 to May 2020. The second to third mature leaves (one bud and two leaves) of each plant’s canopy were dark-adapted for 30 min by the use of leaf clips. Following this, the central region of the adaxial leaf surface was subjected to a saturating light pulse of 3,500 μmol m^− 2^ s^− 1^ (690 nm) prior to being measured. Analysis of photosynthetic capacity and ChlF parameters have been described in our previous paper (Chen et al. 2021). Briefly, Fv/Fm and ΦPSII were calculated as (Fm - Fo) / Fm and (Fm’ - Fo’) / Fm’, respectively. Fo (Fo’) and Fm (Fm’) are the minimal and maximal fluorescence values of dark-adapted and during-illumination leaves, respectively. Values of the Fo and Fm of the dark-adapted samples were determined, and gas exchange and ChlF measurements were simultaneously measured at 10:00 a.m. on a clear day under the stable environmental conditions of the leaf chamber. Environmental conditions during the experiment were set to a gas-flow rate at 750 μmol s^–1^, gas-mixer speed to level 7, assimilator temperature to 25°C, and relative humidity to 75%. ΔF/Fm’ was calculated as (Fm’ - F) / Fm’. ETR was obtained as ΔF/Fm’ x PPFD x 0.5 × 0.84. NPQ was calculated as (Fm/Fm’) -1. Moreover, Rd (*μ*mol CO_2_ m^− 2^ s^− 1^), Qy (CO_2_/ PPFD), and LCP (*μ*mol PPFD m^− 2^ s^− 1^) were obtained from the linear regression of photosynthetic light response curves to illumination measured from 0 ∼ 100 μmol PPFD m^− 2^ s^− 1^. Values for Pn and Gs were simultaneously calculated and recorded inside the chamber of the photosynthesis analyzer (GFS-3000FL, Walz, Effeltrich, Germany). The operation was automatic, and data were stored in the computer within the console and analyzed. All measurements were performed on fifteen leaves (the second to third mature leaves) from five replicates for each treatment of 380–400 ppm in the atmospheric environment at room temperature (25 °C) from mid-morning until mid-afternoon (10:00 ∼ 17:00).

### Statistical analysis

The gas exchange of plant response to different illumination intensities was recorded by the instrument after 5–10 min equilibrium in the chamber and three replications were averaged to presnt in the figure. Statistical analyses were performed using PASW Statistics 18 software (PASW 18, IBM, USA). Gas exchange and ChlF measurements were analyzed using a single-factor analysis of variance (ANOVA) to check for significant differences between CA and SC, and differences among season means were assessed using Tukey’s HSD test with *p* < 0.05 significance. In addition, two-way ANOVA was used for the interaction of tillages and seasons. Multiple comparisons were performed using the least-significant difference. Regression analyses were used to examine relationships between Gs and Pn and among ETR, Pn, and NPQ. In addition, model datasets were based on at least 25 leaves (the second to third mature leaves) from each PPFD level, and ChlF parameters were calculated using ETR data from the model validation datasets. Several models were tested, including linear regression models being selected for the interpretation of the relationship between ChlF parameters and PPFD. All models were evaluated for goodness of fit by the graphical analysis of residuals and by computing correlation coefficients at a significance level of *p* < 0.05 between the gas-exchange and ChlF parameters. The linear regression model performance proved more suitable.

## Results

**Fig. 1:**
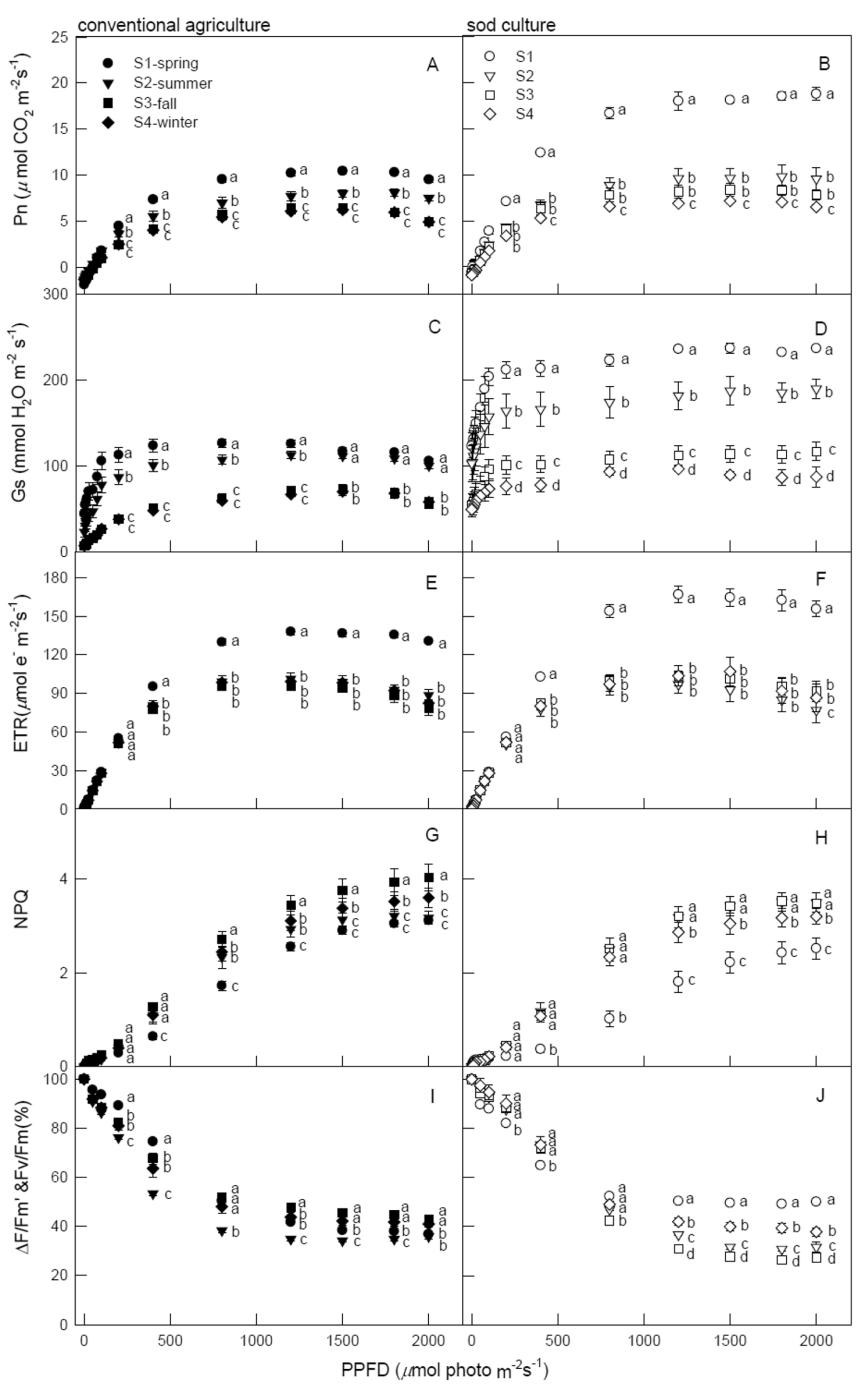
Comparison of conventional agriculture and sod culture on Pn, Gs, ETR, NPQ, ΔF/F’m, and Fv/Fm of tea plants grown in four seasons (●=spring, ▼=summer, ■=fall, ◆=winter, The solid ones are CA and the hollow ones are SC.) monitored from June 2019 to May 2020. Data are mean ± standard error, and each point represents the mean of five mature leaves. Different letters indicate significant differences in Tukey’s HSD analyses over four seasons (*p* < 0.05)

The impact of two cultivation methods and four seasons on tea photosynthetic parameters (Pn, Gs, slope of Gs-Pn, ETR, NPQ, ΔF/Fm’, Fv/Fm, Rd, Qy, LCP, and Amax) under 2,000 PPFD is presented in Table 1. Except for Rd in seasons and Fv/Fm in T x S, all photosynthetic indices exhibited significant differences (*p* < 0.0001, 0.001, 0.05, and 0.01) in both main and interaction effects. Furthermore, Fig. 1 indicates that, under 0-100 PPFD, no significant seasonal differences in any photosynthetic parameters were observed in either tillage. However, for 200-2,000 PPFD, spring Pn values were notably higher than in other seasons, regardless of tillage (Fig. 1A, B), except for 200 PPFD under CA, where no significant seasonal differences in Pn values were noted (Fig. 1A). As light intensity escalated, Pn values under CA rose from 0 to 1,500 PPFD across all seasons, then gradually declined (Fig. 1A), whereas under SC, Pn significantly increased in the order of winter, fall, summer, and spring (Fig. 1B), indicating seasonal Pn responses to all light intensities. Spring Gs content under CA significantly surged from 200 to 1,200 PPFD compared to other seasons (Fig. 1C), whereas under SC, Gs values decreased significantly from spring to winter across 200-2,000 PPFD (Fig. 1D). Spring ETR levels were significantly higher than other seasons from 400 to 2,000 PPFD, and ETR for both tillages consistently rose from 0 to 1,200 PPFD, then declined (Fig. 1E, F). Figure 1G and H show that, regardless of tillage, fall and winter NPQ values from 400 to 2,000 PPFD were significantly higher than in spring, and NPQ increased in all mature leaves, seasons, and tillages as light intensity rose from 0 to 2,000 PPFD. However, as light intensity rose from 0 to 1,200 PPFD, both tillages exhibited a steady decrease in ΔF/Fm’ (%) in all seasons, followed by stabilization from 1,500 to 2,000 PPFD (Fig. 1I, J). Under CA, spring and fall ΔF/Fm’ and Fv/Fm (%) from 200 to 2,000 PPFD were significantly higher than in summer (Fig. 1I). Conversely, under SC, spring ΔF/Fm’ and Fv/Fm (%) from 200 to 400 PPFD and 800-2,000 PPFD were significantly lower and higher, respectively, than in fall (Fig. 1J).

**Table 1:** ANOVA of tillage (T), season (S), and their interactions (T x S) for Pn, Gs, slope of Gs-Pn, ETR, NPQ, ΔF/Fm’ (%), Fv/Fm, Rd, Qy, LCP, and Amax of tea plants at 2,000 *μ*mol m^− 2^ s^− 1^ PPFD under CA and SC in four seasons

	F-value and significance										
Source of variance	Pn	Gs	Slope	ETR	NPQ	ΔF/Fm’ (%)	Fv/Fm	Rd	Qy	LCP	Amax
Tillage (T)	457.66 ****	376.08 ****	39.03 ****	4.70 *	26.82 ****	67.84 ****	26.89 ****	58.93 ****	54.57 ****	181.46 ****	305.79 ****
Season (S)	428.50 ****	1038.31 ****	13.24 ****	67.30 ****	44.44 ****	108.02 ****	14.70 ****	2.014 NS	66.11 ****	15.31 ****	370.91 ****
T × S	93.39 ****	81.00 ****	5.19 **	4.64 **	13.46 ****	9.98 ****	0.46 NS	8.23 ***	4.57 **	7.45 **	84.38 ****

Table 2 presents the variations in Rd, Qy, LCP, Amax, and Fv/Fm of tea mature leaves over eight seasons under two tillages. Regardless of the season, all Rd values in CA (1.23 ∼ 1.86 μmol CO_2_ m^− 2^ s^− 1^) were significantly higher than in SC (0.57 ∼ 1.03 μmol CO_2_ m^− 2^ s^− 1^). Under both tillage methods, Qy significantly increased in spring (0.04 CO_2_/PPFD) compared to other seasons (0.02 ∼ 0.03 CO_2_/PPFD), suggesting that the relative increase in CO_2_ and carbon sink behavior may be a response to physiological acclimation in spring. CA exhibited significantly higher LCP values (39.88 ∼ 61.10 μmol PPFD m^− 2^ s^− 1^) compared to SC (11.66 ∼ 35.05 μmol PPFD m^− 2^ s^− 1^) across seasons in both tillage methods. Moreover, regardless of the tillage method, Amax values significantly increased in the order of spring, summer, fall, and winter. Similarly, Fv/Fm levels significantly increased in spring (0.76) compared to other seasons (0.73 ∼ 0.75) under both tillage methods.

**Table 2:** Influence of CA and SC on Fv/Fm, Rd, Qy, LCP, Amax, and Fv/Fm of tea plants grown in four seasons monitored from June 2019 to May 2020

Photosynthetic parameters	Conventional agriculture (CA)				Sod culture (SC)				
	Spring	Summer	Fall	Winter		Spring	Summer	Fall	Winter
Rd (*μ*mol CO_2_ m^− 2^ s^− 1^)	1.86^a^	1.23^c^	1.49^b^	1.28^c^		0.57^f^	0.76^e^	1.03^d^	1.03^d^
Qy (CO_2_/ PPFD)	0.04^a^	0.03^b^	0.02^c^	0.02^c^		0.04^a^	0.03^b^	0.03^b^	0.03^b^
LCP (*μ*mol PPFD m^− 2^ s^− 1^)	49.38^c^	39.88^d^	61.10^a^	54.03^bc^		11.66^f^	24.17^e^	27.88^e^	35.05^d^
Amax (*μ*mol CO_2_ m^− 2^ s^− 1^)	10.43^b^	8.05^cd^	6.46^e^	6.16^e^		18.82^a^	9.85^b^	8.36^cd^	7.16^d^
Fv/Fm	0.76^a^	0.74^c^	0.74^c^	0.73^d^		0.76^a^	0.75^b^	0.75^b^	0.75^b^

Means followed by same letter within rows of eight seasons under CA and SC methods are not significantly different according to Tukey’s HSD analyses (*p* < 0.05). Each point represents the mean of 5 mature leaves

**Fig. 2:**
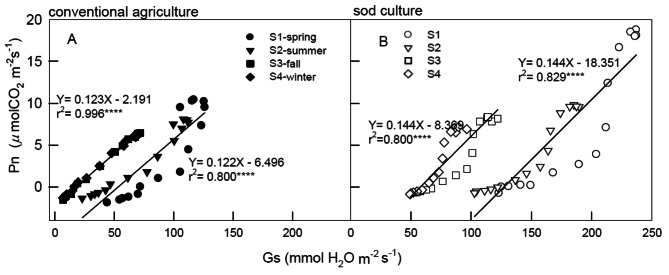
Correlations between the Gs and Pn of tea grown under conventional agriculture (panel **A**) and sod culture (panel **B**) in four seasons (●=spring, ▼=summer, ■=fall, ◆=winter, The solid ones are CA and the hollow ones are SC.) monitored from June 2019 to May 2020. Plants were subjected to light irradiations of 0, 5, 10, 15, 25, 50, 75, 100, 200, 400, 800, 1,200, 1,500, 1,800, and 2,000 *μ*mol m^− 2^ s^− 1^ PPFD for 75 min. Each symbol represents the average of five mature leaves on one plant, and five plant**s** were randomly selected for tillage and season treatments. Each line represents the time point of 48 values (two seasons multiplied by 15 PPFD) from the model’s validation datasets. The correlation coefficients (r) are calculated and significance (*p*) is at the 0.0001 probability (*****) level

Figure 2 illustrates the significant impact of light intensity (ranging from 0 to 2,000 PPFD) and seasonal variation on the correlations between stomatal conductance (Gs) and net photosynthetic rate (Pn) in tea plants under two different tillages, CA and SC In both fall and winter and spring and summer under CA, there were positive and significant correlations between Gs and Pn with r² values of 0.996 and 0.8, respectively (Fig. 2A). Similar significant positive correlations were observed under SC, with r² values of 0.8 and 0.829 for fall and winter and spring and summer, respectively (Fig. 2B). Interestingly, a higher slope in the Pn/Gs linear relationship, equivalent to WUE, was detected in fall and winter under SC (0.144) compared to CA (0.122 and 0.123), indicating higher photosynthesis in SC treatments.

**Fig. 3:**
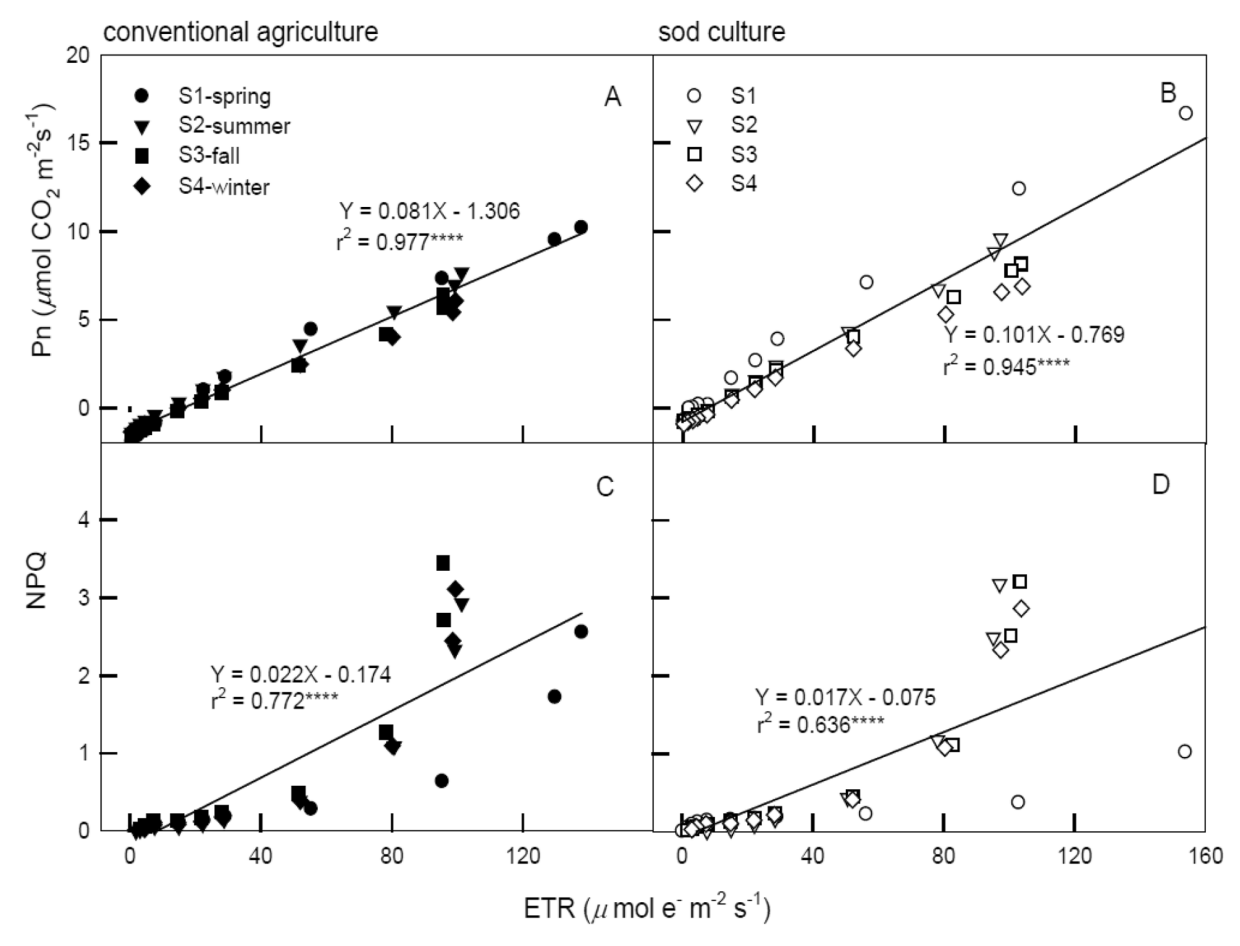
Correlations among ETR and Pn, NPQ of tea plants grown under conventional agriculture (panels **A**, **C**) and sod culture (panels **B**, **D**) in four seasons (●=spring, ▼=summer, ■=fall, ◆=winter, The solid ones are CA and the hollow ones are SC.) monitored from June 2019 to May 2020. Plants were subjected to light irradiances at 0, 5, 10, 15, 25, 50, 75, 100, 200, 400, 800, and 1,200 *μ*mol m^− 2^ s^− 1^ PPFD for 60 min. Each symbol represents the average of five mature leaves on one plant, and five plant**s** were randomly selected from tillage and season treatments. Each line represents the time point of 48 values (four seasons multiplied by 12 PPFD) from model validation datasets. Correlation coefficients (r) are calculated and significance (*p*) is at the 0.0001 probability (*****) level

Figure 3 depicts the relationships among ETR, Pn, and NPQ of plants under CA and SC across four seasons at light intensities ranging from 0 to 1,200 μmol m^− 2^ s^− 1^ PPFD. Significant correlations were found between ETR and Pn (r² = 0.977 and 0.945 for CA and SC, respectively; Fig. 3A, B) and between ETR and NPQ (r² = 0.772 and 0.636 for CA and SC, respectively; Fig. 3C, D) under the same PPFD conditions and seasons.

**Fig. 4:**
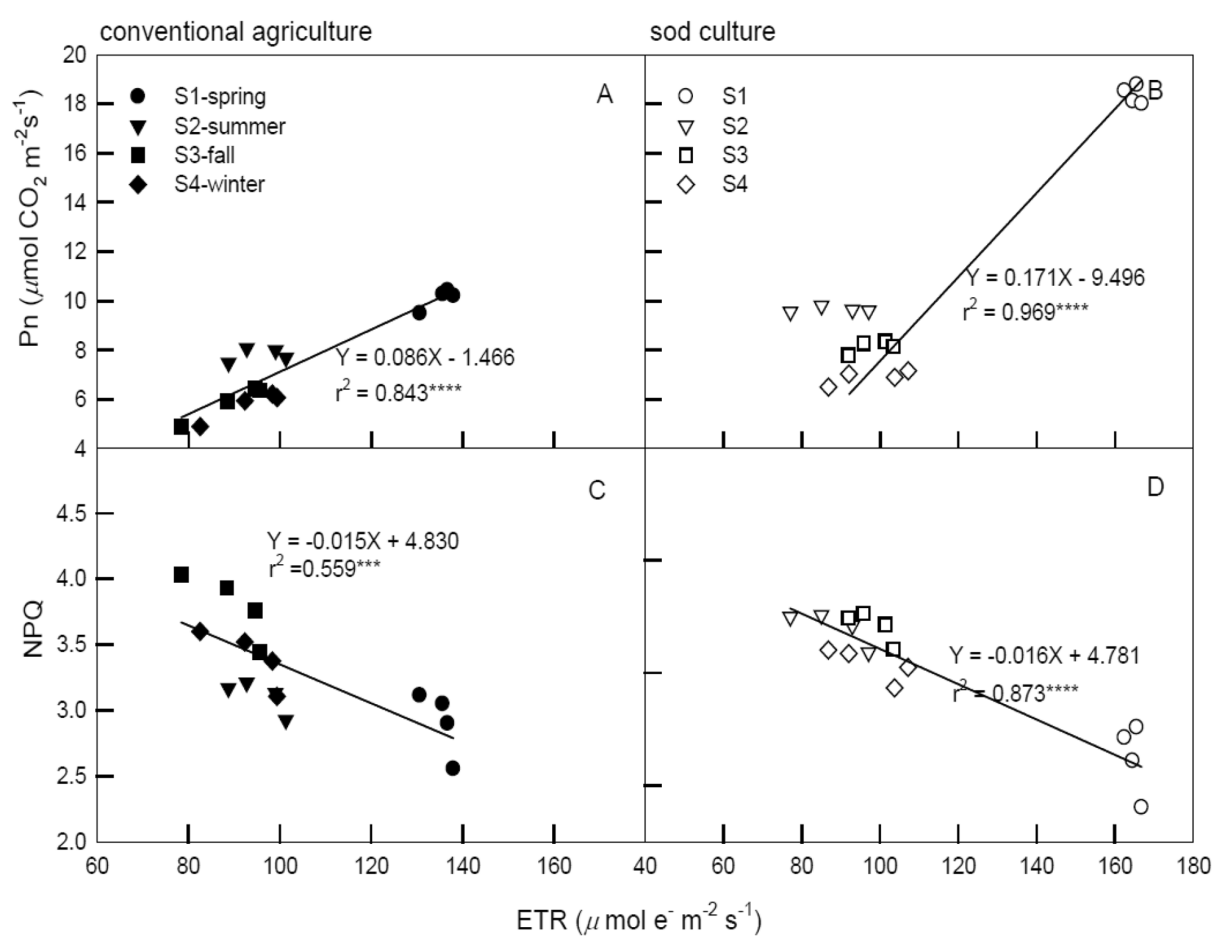
Correlations among ETR and Pn, NPQ of tea plants grown under conventional agriculture (panels **A**, **C**) and sod culture (panels **B**, **D**) in four seasons (●=spring, ▼=summer, ■=fall, ◆=winter, The solid ones are CA and the hollow ones are SC.) monitored from June 2019 to May 2020. Plants were subjected to light irradiances at 1,200, 1,500, 1,800, and 2,000 *μ*mol m^− 2^ s^− 1^ PPFD for 20 min. Each symbol represents the average of five mature leaves on one plant, and five plant**s** were randomly selected from tillage and season treatments. Each line represents the time point of 16 values (four seasons multiplied by 4 PPFD) from model validation datasets. Correlation coefficients (r) are calculated and significance (*p*) is at the 0.0001 probability (*****) level

Figure 4 illustrates the correlations among ETR, Pn, and NPQ in tea grown under CA and SC at higher light intensities (1,200 to 2,000 μmol m^− 2^ s^− 1^ PPFD). ETR was significantly and positively correlated with Pn under both CA and SC (r² = 0.843 and 0.969, respectively; Fig. 4A, B), indicating notably higher Pn values in spring under SC compared to CA. This suggests a stronger photosynthesis rate in SC mature leaves during spring. Conversely, ETR values were significantly and negatively correlated with NPQ (r² = 0.559 and 0.873 for CA and SC, respectively; Fig. 4C, D). Additionally, higher NPQ values were observed in spring compared to other seasons under both tillage methods, indicating a stronger non-photochemical dissipation ability in spring relative to other seasons.

## Discussion

The ChlF components of tea plants were utilized to assess various functional levels of photosynthesis induced by light intensities and tillage methods. Figure 1 demonstrates that, at light intensities ranging from 800 to 2,000 PPFD in spring, the values of net Pn, Gs, ETR, and ΔF/Fm’ and Fv/Fm (%) for SC were comparatively higher than those for conventional agriculture (CA), while NPQ values for SC were relatively lower than those for CA. This suggests that SC experienced less photoinhibition in spring, maintaining high photochemical efficiency and photosynthesis rates. With increasing light intensity, higher ETR values during spring correlated with higher photosynthetic efficiency compared to other seasons, suggesting that tea plants adapt to high light intensities in spring. Furthermore, when light intensity was maintained below 1,500 PPFD, Pn, Gs, ETR, and NPQ all increased simultaneously, indicating that excess light energy potentially dissipated via heat quenching, and that there exists an optimal light intensity (1,200-1,500 PPFD) for tea plant growth.

Comparatively lower NPQ levels were observed in both CA and SC during spring (Fig. 1G,H), while Pn at 1,200-1,500 PPFD was highest, reaching 10.4 μmol CO_2_ m^− 2^ s^− 1^ for CA and 18.1 μmol CO_2_ m^− 2^ s^− 1^ for SC (Fig. 1A,B). This implies that higher photosynthesis rates consume more light energy, reducing excess light energy, and, consequently, resulting in lower NPQ and photoinhibition (Fig. 1I, J) (Demmig-Adams et al., 2006; Wong et al., 2016; Demmig-Adams et al. 2020). Therefore, higher Pn, Gs, and ETR values detected in spring than in other seasons might be caused by younger leaves in spring. Additionally, the average high temperatures of 35 °C in summer and 30 °C in fall in Taiwan may contribute to higher NPQ performances (Fig. 1G, H) and lower Pn levels (Fig. 1A, B) for both CA and SC compared to spring. Generally, high temperature and high light intensity negatively affect photosynthetic capacity, and high temperatures usually coincide with high light intensities. In photosynthetic organisms, elevated irradiance levels during environmental stress conditions, such as high temperatures, frequently lead to photoinhibition, characterized by a reduction in photosynthetic activity (Pn). This phenomenon occurs due to the surplus light energy exacerbating the generation of detrimental reactive oxygen species within the chloroplasts (Endo et al. 2023). PSII is often the most sensitive component of the photosynthetic apparatus to high temperatures or light intensities and is susceptible to photodamage (Pospíšil, 2016; Gao et al., 2019). This susceptibility may lead to higher photoinhibition (Murata et al., 2007; Zulfugarov et al. 2007; Tikkanen et al., 2014) and lower Pn (Molina-Bravo et al. 2011) in tea plants during summer and fall.

Increasing light intensity was associated with a rise in NPQ and a decline in ΔF/Fm’ (Fv/Fm) across all seasons and cultivations (Fig. 1I, J), suggesting that both tillage methods resulted in low photosynthetic rates, necessitating the dissipation of excess energy to safeguard the PS. At light intensities of 1,200 to 2,000 PPFD, NPQ and ΔF/Fm’ (Fv/Fm) values were consistently maintained at high and low levels, respectively, with minimal or no variation, signifying that their photochemical capacity had reached a plateau. The diminished ΔF/Fm’ (Fv/Fm) levels (below 60%) observed at 800 to 2,000 PPFD, and the reduced Pn (Fig. 1A, B) and ETR (Fig. 1E, F) levels observed at 1,800 to 2,000 PPFD, indicate the presence of photoinhibition. Regardless of the season or cultivation method, a light intensity ranging from 800 to 1,500 PPFD was deemed optimal for plant growth. Thus, management strategies for tea plants should aim to mitigate the effects of high temperatures and intense light during summer and fall, for instance, by spraying water in the afternoon or using the surrounding terrain and trees to shield against excessive incident light, ultimately enhancing tea leaf production and quality. Moreover, SC outperformed CA as SC bolstered soil organic matter content, augmented soil water-holding capacity, and ameliorated the garden microclimate (Bai et al. 2017; Lin et al., 2019), thereby rendering SC more resilient to the climatic extremes associated with climate change.

In Table 2, the Rd and LCP values under CA were significantly higher than those under SC, potentially due to the long-term tillage cropping system and the year-round reduction in soil moisture content (Dominika et al. 2020) at the study sites, which subsequently led to reduced foliar respiration, growth rates, and carbon accumulation potential (Chen et al. 2021). This relative water deficit might have induced a drought-like state in CA-treated tea plants, resulting in elevated Rd and LCP values. In contrast, the ground surface cover in the SC conservation agricultural system likely mitigated water evaporation, enhanced WUE, improved leaf morphology and photosynthetic properties, increased soil organic content, and boosted carbon sequestration, especially in the soil surface layer. Notably, the levels of Qy, Amax, and Fv/Fm in mature leaves during spring were significantly higher than in other seasons, suggesting that the precipitation and temperature in spring were favorable for tea plant growth. Specifically, CA employed an automatic watering system, whereas SC relied solely on natural precipitation for water supply. The photosynthetic light response curve not only illustrated the expected relationship between light intensity and leaf Pn but also indicated that the calculated parameters LCP, Amax, and Rd could be employed to assess the impact of soil conditions and climatic factors on plants (Lachapelle et al., 2012; Lang et al. 2013; Chen et al. 2018). These parameters are also linked to metabolic changes that can be utilized to predict the effects of future climate change on plant productivity (O’Leary et al., 2017). In our study, Rd, Qy, LCP, Amax, Fv/Fm, ΔF/Fm’, Pn, Gs, ETR, and NPQ were found to be appropriate for evaluating photosynthetic efficiency. Over time, SC is likely to enhance biodiversity (Trifonova et al. 2022), optimize the microclimate (Liu et al. 2021), and lead to improved Qy, LCP, Amax, and Fv/Fm in mature leaves exposed to varying light levels. Consequently, SC could bolster the resilience of tea fields to climate change, thereby sustaining tea production and economic revenue. SC may be particularly advantageous for ecosystems with uneven rainfall distribution.

The comparison between CA and SC across different seasons serves as a valuable tool for evaluating the impacts of global climate change and its applicability to the physiological states of crops. Throughout all seasons and light illuminations, the photosynthesis curves of CA and SC displayed positive and significant correlations between Pn and Gs (Fig. 2), indicating that the increase in Pn might be attributed to stomatal opening, which in turn limits the reduction of photosynthetic rates. During the photosynthesis saturation period, changes in Pn were primarily influenced by Gs, subsequently leading to the maintenance of high WUE. Generally, Gs levels in spring and summer were significantly higher than those in fall and winter, suggesting that the stomatal opening speed of tea plants was not restricted during suitable temperatures, resulting in higher Pn and a more efficient water-use strategy developed in response to spring and summer conditions. CA exhibited lower water efficiency in spring and summer due to the ease of surface water evapotranspiration, which led to reduced Pn. Despite the presence of a sprinkler irrigation system in the field, water shortages were encountered, particularly during the absence of rainfall in spring and summer throughout the experimental period. The elevated Pn and Gs in spring and summer might also be attributed to the new leaves being well-suited to the temperatures. Gs levels under CA were consistently lower than those under SC in each season, which might have constrained tea leaf photosynthesis, placing CA in a potentially water-stressed condition. Since photosynthesis, CO_2_, and water are pivotal for plant growth and yield, plants must strike a balance between CO_2_ uptake for photosynthesis and water loss through transpiration. Stomata regulate gas exchange between the leaf interior and external atmosphere, thereby enhancing WUE (Deans et al. 2018; Eyland et al. 2021). ETR exhibited significant and positive correlations with Pn and NPQ when exposed to 0 ∼ 1,200 PPFD (Fig. 3) and 1,200 ∼ 2,000 PPFD (Fig. 4A, B), whereas significant and negative correlations were observed between ETR and NPQ at high illuminations of 1,200 ∼ 2,000 PPFD (Fig. 4C, D). These results suggest that photochemical and non-photochemical quenching would concurrently up-regulate Pn at 0 ∼ 1,200 PPFD. Photoinhibition might occur when tea plants are intolerant to high light, and the elevated photosynthesis in spring could be attributed to its temperature factor. However, at 1,800 ∼ 2,000 PPFD, photoinhibition occurred in PSII), leading to decreased photochemical efficiency, ETR, and Pn. As a result, tea plants might be experiencing high-illumination stress, causing a reduction in ETR (Fig. 1E, F). Nevertheless, the excess light energy generated would consequently maintain NPQ at 1,200 ∼ 2,000 PPFD (Fig. 2G, H) due to the continuous photoprotective mechanism maintained by a high proportion of NPQ as illumination increased.

The photosynthetic parameters analyzed in this study are highly sensitive indicators that enable quick identification of the physiological status of plants (Hirotsu et al. 2005). Specifically, ΔF/Fm’ (Fv/Fm) represents the photosynthetic potential for photochemical dissipation, and the photochemical ability of photosystem II (PSII) under various light intensities exhibits a linear relationship with the CO_2_ fixation rate (Cui et al. 2006). High irradiation exposure may significantly depress ΔF/Fm’ (Fv/Fm), leading to the suppression of the electron transfer chain (Wu et al. 2015). The ETR is valuable for non-destructively estimating net photosynthesis rate (Pn) and NPQ, thus simplifying evaluations of the relationship between heat dissipation and photosynthetic efficiency. These parameters are expected to exhibit quantifiable differences between CA and SC tea plant cultures under various seasons and light intensities. Moreover, these metrics should be capable of indicating how controlled light intensities might be utilized to enhance rapid, large-scale, and precise commercial management of tea plants. Given that ΔF/Fm’ (Fv/Fm) encompasses all these parameters, its use is recommended across all tea regions for evaluating tea plants in terms of radiation use efficiency and photosynthetic system status. Understanding the photosynthetic characteristics of tea plants under different seasons and tillage methods, through remote sensing of their physiological state, would undoubtedly inform field cultivation management. For example, optimizing field tillage methods and implementing artificial shading could help avoid photoinhibition factors that are anticipated to intensify due to global climate change. Such remote sensing is expected to be particularly beneficial in fields experiencing seasonal aridity during cycles of prolonged drought and heavy rain.

## Conclusions

The positive effects of SC on Amax, Fv/Fm, Gs, WUE, ETR, and NPQ suggest that SC is advantageous for the production and drought resistance of tea plants under future climate change scenarios. Quantifying the responses of Pn, Amax, Fv/Fm, Gs, WUE, ETR, and NPQ to seasonal light variations is crucial for developing indicators for tillage management. Utilization of parameters such as Amax, WUE, ETR, and NPQ not only facilitates the rapid assessment of the photosynthetic capacity of tea plants across four seasons, taking into account responses to factors like light intensity, drought, and temperature, but also allows for accurate field management aligned with the environmental benefits of SC. This approach provides a tea garden management model that addresses the impacts of climate change.

### Electronic Supplementary Material

Below is the link to the electronic supplementary material.

Supplementary Material 1

## Data Availability

The data and materials are available upon reasonable request from the corresponding author.

## Abbreviations

Amax: Maximum net assimilation of CO_2_
CA: Conventional agriculture
ETR: Electron transport rate
F/Fm’: Actual quantum efficiency of PSII
Fv/Fm: Maximal quantum yield of PSII photochemistry
Gs: Stomatal conductance
LCP: Light compensation point
LHC: Light-harvesting complexes
NPQ: Nonphotochemical quenching
Pn: Net photosynthesis rate
PPFD: Photosynthetic photon flux densities
PS: Photosystems
Qy: Light quantum yield of CO_2_
Rd: Dark respiration rate of CO_2_
SC: Sod culture
WUE: Water use efficiency

## References

[CR1] Agriculture and Food Agency, COA (2023) COA Tea (*Camellia sinensis* L.) production survey in Taiwan https://www.afa.gov.tw/cht/index.php?act=article&code=print&ids=307&article_id=45522&flag=detail. (accessed on 12 Dec 2023)

[CR3] Bai R, Wang Y, Ma Z, Yun-yun J (2017). Research Advance on Sod Culture in Peach Orchard. J Agric Sci Technol.

[CR5] Chen CI, Wang YN, Yu JC (2018). Diurnal and seasonal CO_2_ assimilation by four Plantation species in Taiwan. Sci.

[CR6] Chen CI, Lin KH, Huang MY, Yang CK, Lin YH, Hsueh ML, Lee LH, Wang CW (2021). Photosynthetic physiology comparisons between no tillage and Sod Culture of Citrus Farming in different Seasons under various light intensities. Agronomy.

[CR7] Cui L, Li J, Fan Y, Xu S (2006). High Temperature effects on Photo-Synthesis, PSII functionality and antioxidant activity of two *Festuca arundinacea* cultivars with different heat susceptibility. Bot Stud.

[CR8] Dai W, Qi D, Yang T, Lv H, Guo L, Zhang Y, Zhu Y, Peng Q, Xie D, Tan J, Lin Z (2015). Nontargeted analysis using Ultraperformance liquid chromatography-quadrupole time-offlight mass spectrometry uncovers the effects of harvest season on the metabolites and taste quality of tea (*Camellia sinensis* L). J Agric Food Chem.

[CR10] Deans RM, Brodribb TJ, Busch FA, Farquhar GD (2018). Plant water-use strategy mediates stomatal effects on the light induction of photosynthesis. New Phytol.

[CR1100] Demmig-Adams B, Ebbert V, Mellman DL. Mueha KE, Schaffera L, Funkb C, Zartera R, Adamskac I, Janssond S, Adams III WW (2006) Modulation of PsbS and flexible vs sustained energy dissipation by light environment in different species. Physiologia Plantarum 127:670–680. 10.1111/j.1399-3054.2006.00698.x

[CR11] Demmig-Adams B, Stewart JJ, López-Pozo M, Polutchko SK, William AIII (2020). Zeaxanthin, a molecule for Photoprotection in many different environments. Molecules.

[CR12] Dominika K, Natacha B, Helene Bracht J, Jaak T, Klaus B, Katarina H, Paul M, Andreas F (2020). Effects of simulated drought on biological soil quality, microbial diversity and yields under long-term conventional and organic agriculture. FEMS Microbiol Ecol.

[CR13] Endo H, Moriyama H, Okumura Y (2023). Photoinhibition and Photoprotective responses of a Brown Marine Macroalga Acclimated to different light and nutrient regimes. Antioxidants.

[CR14] Eyland D, van Wesemael J, Lawson T, Carpentier S, Notes A (2021). The impact of slow stomatal kinetics on photosynthesis and water use efficiency under fluctuating light. Plant Physiol.

[CR15] Feng YL, Cao KF, Feng ZL (2002). Thermal dissipation, leaf rolling and inactivation of PSII reaction centres in *Amomum Villosum*. J Trop Ecol.

[CR150] Ferdous Z, Zulfiqar F, Datta A, Hasan AK, Sarker A (2021) Potential and challenges of organic agriculture in Bangladesh: a review. J Crop Improv 35:403–426. 10.1080/15427528.2020.1824951

[CR1500] Gao YB, Zheng WW, Zhang C, Zhang LL, Xu K (2019) High temperature and high light intensity induced photoinhibition of bayberry (Myrica rubra Sieb. et Zucc.) by disruption of D1 turnover in photosystem II. Hortic Sci 248:132–137. 10.1016/j.scienta.2019.01.007

[CR16] Hayat K, Iqbal H, Malik U, Bilal U, Mushtaq S (2015). Tea and its consumption: benefits and risks. Crit Rev Food Sci Nutr.

[CR28] Hirotsu N, Makino A, Yokota S, Expand A (2005). The Photosynthetic properties of Rice leaves treated with low temperature and high irradiance. Plant Cell Physiol.

[CR17] International Tea Committee (2021) Annual bulletin of statistics. https://inttea.com/publications. (accessed on 14 Feb 2022)

[CR29] Kałuzewicz A, Bączek-Kwinta R, Krzesi´nski W, Spiżewski T (2018). Effect of biostimulants on chlorophyll fluorescence parameters of broccoli (*Brassica oleracea* var. *italica*) under drought stress and rewatering. Acta Sci Pol Hortorum Cultus.

[CR30] Kfoury N, Morimoto J, Kern A, Scott ER, Orians CM, Ahmed S, Griffin T, Cash SB, Stepp JR, Xue D, Long C, Robbat A (2018). Striking changes in tea metabolites due to elevational effects. Food Chem.

[CR18] Lachapelle PP, Shipley B (2012). Interspecific prediction of photosynthetic light response curves using specific leaf mass and leaf nitrogen content: effects of differences in soil fertility and growth irradiance. Ann Bot.

[CR20] Lang Y, Wang M, Zhang GC, Zhao QK (2013). Experimental and simulated light responses of photosynthesis in leaves of three tree species under different soil water conditions. Photosynthetica.

[CR21] Lin YH, Chiu JY (2019). Studies on the sod culture and the management of soil moisture for the improvement of wax apple quality. Afr J Biotechnol.

[CR22] Liu S, Gao J, Chen Z, Qiao X, Huang H, Cui B, Zhu Q, Dai Z, Wu H, Pan Y, Yang C, Liu J (2017). Comparative proteomics reveals the physiological differences between winter tender shoots and spring tender shoots of a novel tea (*Camellia sinensis* L.) cultivar evergrowing in winter. BMC Plant Biol.

[CR23] Liu YP, Mao YF, Hu YL, Zhang LL, Yin YJ, Pang HL, Su XF, Yang L, Shen X (2021). Effects of grass planting in apple orchard on soil microbial diversity, enzyme activities and carbon components. J Plant Nutr Fertil.

[CR24] Molina-Bravo R, Arellano C, Sosinski BR, Sosinski BR (2011). A protocol to assess heat tolerance in a segregating population of raspberry using chlorophyll fluorescence. Sci Hortic.

[CR26] Moya I, Loayza H, Lopez ML, Quiroz R, Ounis A, Goulas Y (2019). Canopy chlorophyll fluorescence applied to stress detection using an easy to build microlidar. Photosynth Res.

[CR2600] Murata N, Takahashi S, Nishiyama Y, Allakhverdiev SI (2007) Photoinhibition of photosystem II under environmental stress. Biochim. Biophys. Acta - Bioenerg. 1767:414–421. 10.1016/j.bbabio.2006.11.019 10.1016/j.bbabio.2006.11.019 17207454

[CR25] Murchie EH, Niyogi KK (2010). Manipulation of Photoprotection to Improve Plant Photosynthesis. Plant Physiol.

[CR32] O’Leary BM, Lee CP, Atkin OK, Cheng R, Brown TB, Millar AH (2017). Variation in Leaf respiration rates at Night correlates with carbohydrate and amino acid supply. Plant Physiol.

[CR33] Portela FCS, Macieira BPB, Zanetti LV, Gama VN, Silva DM, Dias Milane CR, Cuzzuol G (2019). How does *Cariniana estrellensis* respond to different irradiance levels?. J Res.

[CR3400] Pospíšil P (2016) Production of reactive oxygen species by photosystem ii as a response to light and temperature stress. Front Plant Sci. 7:1950. 10.3389/fpls.2016.01950 10.3389/fpls.2016.01950 PMC5183610 28082998

[CR340] Riedo J, Wettstein FE, Rösch A, Herzog C, Banerjee S, Büchi L, Charles R, Wächter D, Martin-Laurent F, Bucheli TD, Walder F, van der Heijden MGA (2021) Widespread occurrence of pesticides in organically managed agricultural soils—the ghost of a conventional agricultural past? J Environ Sci 55:2709–3452. 10.1021/acs.est.0c06405 10.1021/acs.est.0c06405 33534554

[CR35] Souza CSCR, Santos VAHF, Ferreir MJ, Gonçalves JFC (2017). Biomass, growth and ecophysiological responses of young plants of *Bertholletia excelsa* Bonpl. Subjected to different levels of irradiance. Ciênc Florest.

[CR3600] Tikkanen M, Mekala NR, Aro EM (2014) Photosystem II photoinhibition-repair cycle protects Photosystem I from irreversible damage. Biochim. Biophys. Acta - Bioenerg. 1837:210–215. 10.1016/j.bbabio.2013.10.001 10.1016/j.bbabio.2013.10.001 24161359

[CR36] Trifonova T, Mishchenko N, Shoba S, Bykova E, Shutov P, Saveliev O, Repkin R (2022). Soil and Vegetation Cover and Biodiversity Transformation of Postagrogenic soils of the Volga-Oka Interstream Area. Agronomy.

[CR37] Wang P, Wang Y, Wu QS (2016). Effects of soil tillage and planting grass on arbuscular mycorrhizal fungal propagules and soil properties in citrus orchards in southeast China. Soil Tillage Res.

[CR38] Wang CW, Wong SL, Liao TS, Weng JH, Chen MN, Huang MY, Chen CI (2021) Photosynthesis in response to salinity and submergence in two Rhizophoraceae mangroves adapted to different tidal elevations. Tree Physiol tpab 167. 10.1093/treephys/tpab167 10.1093/treephys/tpab167 34918132

[CR39] Wen B, Ren S, Zhang Y, Duan Y, Shen J, Zhu X, Wang Y, Ma Y, Zou Z, Fang W (2020). Effects of geographic locations and topographical factors on secondary metabolites distribution in green tea at a regional scale. Food Control.

[CR400] Wong SL, Chen CW, Weng JH (2016) Energy quenching and photoinhibition during light induction in four fern species. TW J. Biodivers 18:173–189

[CR40] Wu CW, Lin KH, Lee MC, Peng YL, Chou TY, Chang YS (2015). Using chlorophyll fluorescence and vegetation indices to predict the timing of Nitrogen demand in Pentas lanceolata. Korean J Hortic Sci Technol.

[CR41] Xiang P, Qiufang Z, Marat T, Cheng B, Tan M, Liu J, Wang X, Huang J, Gao S, Lin D, Zhang Y, Wu L, Lin J (2021). Light control of catechin accumulation is mediated by photosynthetic capacity in tea plant (*Camellia sinensis*). BMC Plant Biol.

[CR42] Zeng L, Watanabe N, Yang Z (2019). Understanding the biosyntheses and stress response mechanisms of aroma compounds in tea (*Camellia sinensis*) to safely and effectively improve tea aroma. Crit Rev Food Sci.

[CR45] Zhang Q, Shi Y, Ma L, Ma L (2014). Metabolomic analysis using ultraperformance liquid chromatography-quadrupole-time of flight mass spectrometry (UPLC-Q-TOF MS) uncovers the effects of light intensity and temperature under shading treatments on the metabolites in tea. PLoS ONE.

[CR43] Zhang C, Suen CLC, Yang C, Quek SY (2018). Antioxidant capacity and major polyphenol composition of teas as affected by geographical location, plantation elevation and leaf grade. Food Chem.

[CR46] Zhang L, Cao QQ, Granato D, Xu YQ, Ho CT (2020) Association between chemistry and taste of tea: a review. Trends Food Sci Technol 101:139–149. 10.1016/j.tifs.2020.05.015

[CR44] Zhang Q, Hu J, Liu M, De Vos RCH, Ruan J (2020). Stimulated biosynthesis of delphinidin-related anthocyanins in tea shoots reducing the quality of green tea in summer. J Sci Food Agric.

[CR47] Zou YN, Chen X, Srivastava A, Wang P, Xiang L, Wu QS (2016). Changes in rhizosphere properties of trifoliate orange in response to mycorrhization and sod culture. Appl Soil Ecol.

[CR48] Zulfugarov IS, Ham OK, Mishra S, Kim JY, Nath K, Koo HY, Kim HS, Moon YH, An G, Lee CH (2007). Dependence of reaction center-type energy-dependent quenching on photosystem II antenna size. Biochim Biophys Acta-Bioenerg.

