# Supplementary material for: Gas exchange and chlorophyll fluorescence responses of Camellia sinensis grown under various cultivations in different seasons
Source: Bot Stud. 2024 Mar 22;65:10. doi: 10.1186/s40529-024-00416-0 (PMC10957798; doi:10.1186/s40529-024-00416-0)
Supplement: Supplementary file 1 — Supplementary Material 1 [file 40529_2024_416_MOESM1_ESM.docx]

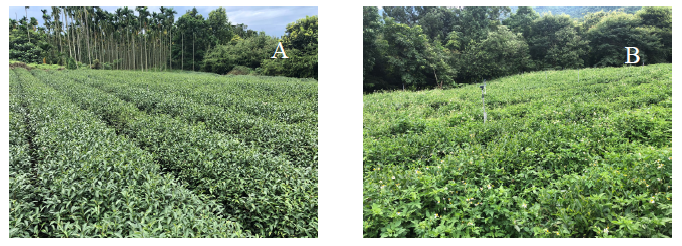


**Figure S1.** The conventional agriculture (panel A) and sod culture (panel B) farming of 20-25 year-old tea plants (Camellia sinensis) 45-55 cm high.

**
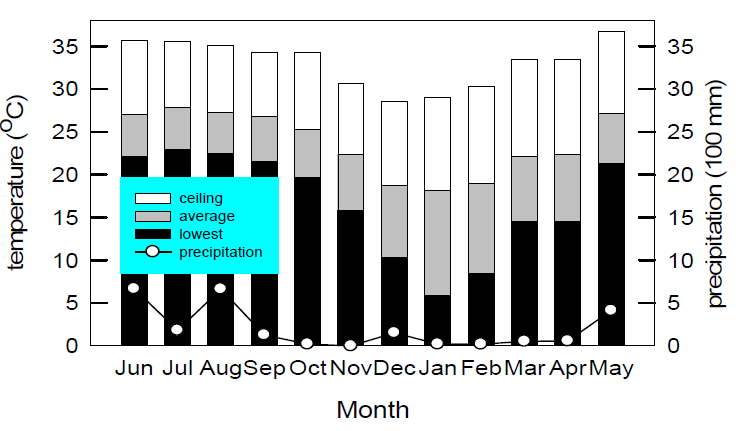
**

**Figure S2.** Monthly air temperatures (bar) and precipitation (circle) during the study period from June 2019 to May 2020 at the lower-elevation mountain area of central Taiwan (120°39'12.4"E, 23°38'36.7"N).
